# Multiple functions of heterogeneous nuclear ribonucleoproteins in the positive single-stranded RNA virus life cycle

**DOI:** 10.3389/fimmu.2022.989298

**Published:** 2022-09-02

**Authors:** Jingming Wang, Di Sun, Mingshu Wang, Anchun Cheng, Yukun Zhu, Sai Mao, Xuming Ou, Xinxin Zhao, Juan Huang, Qun Gao, Shaqiu Zhang, Qiao Yang, Ying Wu, Dekang Zhu, Renyong Jia, Shun Chen, Mafeng Liu

**Affiliations:** ^1^ Institute of Preventive Veterinary Medicine, Sichuan Agricultural University, Chengdu City, China; ^2^ Key Laboratory of Animal Disease and Human Health of Sichuan Province, Sichuan Agricultural University, Chengdu City, China; ^3^ Avian Disease Research Center, College of Veterinary Medicine, Sichuan Agricultural University, Chengdu City, China

**Keywords:** host-pathogen interaction, positive single-stranded RNA virus, heterogeneous nuclear ribonucleoprotein, viral life cycle, immune response

## Abstract

The heterogeneous nuclear ribonucleoproteins (hnRNPs) are a diverse family of RNA binding proteins that are implicated in RNA metabolism, such as alternative splicing, mRNA stabilization and translational regulation. According to their different cellular localization, hnRNPs display multiple functions. Most hnRNPs were predominantly located in the nucleus, but some of them could redistribute to the cytoplasm during virus infection. HnRNPs consist of different domains and motifs that enable these proteins to recognize predetermined nucleotide sequences. In the virus-host interactions, hnRNPs specifically bind to viral RNA or proteins. And some of the viral protein-hnRNP interactions require the viral RNA or other host factors as the intermediate. Through various mechanisms, hnRNPs could regulate viral translation, viral genome replication, the switch of translation to replication and virion release. This review highlights the common features and the distinguish roles of hnRNPs in the life cycle of positive single-stranded RNA viruses.

## Introduction

Positive single-stranded RNA viruses include a broad group of well-known pathogens in the *Picornaviridae*, *Flaviviridae*, *Coronaviridae* and other viral families (1). These viruses generally endanger human health and cause economic burdens as well as societal costs. For example, severe acute respiratory syndrome-coronavirus-2 (SARS-CoV-2, belonging to the *Coronaviridae* family) has spread worldwide for the past two years, threatening lives by causing severe symptoms in patients and resulting in millions of deaths (2). Hepatitis C virus (HCV, belonging to the *Flaviviridae* family) was estimated to infect 71 million people worldwide, and the number of infected people has increased by nearly 2 million a year, causing cirrhosis, hepatocellular carcinoma, liver failure and even death (3, 4). And enterovirus (belonging to the *Picornaviridae* family) threats human health by its extensive outbreak and causing deaths (5). Positive single-stranded RNA viruses mostly contain a limited-sized genome that encodes several or at most dozens of proteins (6). Viruses require assistance from host factors to replicate successfully in cells and also develop diverse mechanisms to exploit host factors to aid the different life cycle stages for maintaining viral efficient propagation (6–8). Positive single-stranded RNA viruses can translocate host factors to the cytoplasm and support their life cycle (9–11). And some proteins in host cells are closely related to viral proteins or RNAs to inhibit virus propagation (12). A recent study revealed that 104 host proteins could interact with SARS-CoV-2 RNA and participate in viral translational initiation, transcription and immune response. Additionally, 23 of these proteins could be targeted with existing drugs (13). To defend themselves from virus infection, host cells also develop some strategies to drive proteins or other host factors to confine viral proteins or RNA to restrain the virus replication (14). Therefore, studying host proteins that interact with viral genomics or viral proteins is beneficial for understanding RNA virus pathogenesis and providing information on developing antiviral therapies and vaccines (15).

HnRNPs constitute a group of RNA-binding proteins that recognize specific RNA sequences and are reported to be frequently involved in RNA metabolism processes such as pre-mRNA splicing, transcription and translation regulation (16). The hnRNP family mainly comprises 20 proteins, and they are named in alphabetical order from hnRNP A1 to hnRNP U (and RALY, which is also known as HNRPCL2 or P542), with molecular weights ranging from 34 kDa to 120 kDa (16). HnRNPs can bind to heterogenous nuclear RNAs (hnRNAs) or pre-mRNAs, which are primary transcripts generated by polymerase II (17). This binding activity is linked with pre-mRNA splicing, causing impaired binding capacity of hnRNP A, B, C and I and leading to splicing inhibition (18). Heterogeneous ribonucleoproteins (hnRNPs) are proteins identified to associate with the virus components during positive-strand RNA virus infection (19–21).

**Figure 1:**
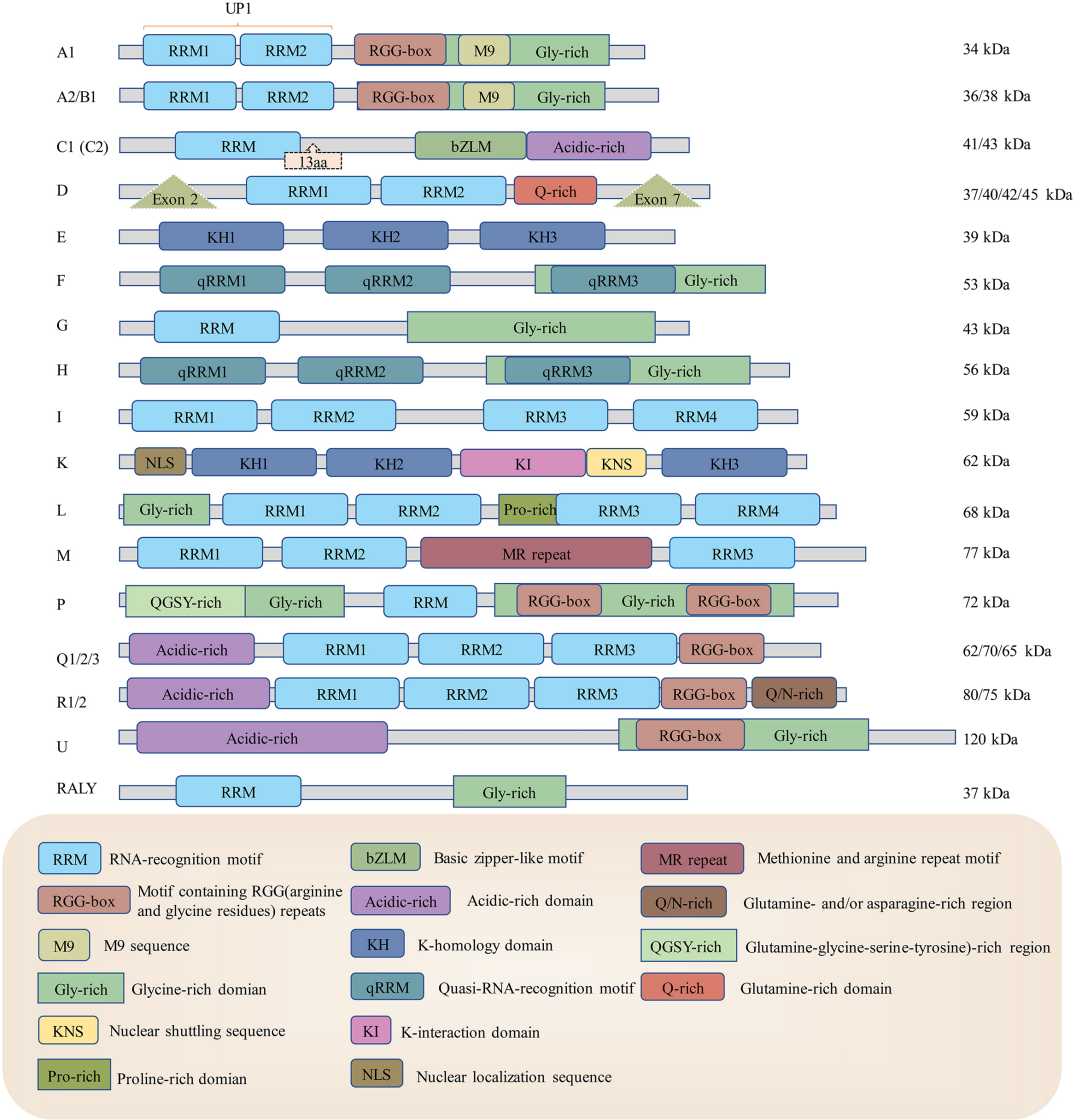
The structures of heterogeneous ribonucleoproteins from hnRNP A1 to RALY. HnRNPs have different structures using some shared and distinctive elements. RRM: RNA recognition motif, KH: K-homology domain, RGG-box: motifs containing arginine and glycine repeats, M9: M9 sequence, Gly-rich: glycine-rich domain, bZLM: basic leucine zipper-like motif, Acidic-rich: acidic-rich domain, Q-rich: Glutamine-rich domain, Exon: The splicing site of enzyme to create various mRNAs, therefore translated into different proteins, NLS: nuclear localization sequence, KI: K-interaction domain, Pro-rich: Proline-rich domain, KNS: nuclear shuttling domain, MR-repeat: methionine and arginine repeat motif, QGSY-rich: (glutamine-glycine-serine-tyrosine)-rich region, Q/N-rich: glutamine- and/or asparagine-rich region. RRMs and KH domains are usually responsible for virus RNA recognition and binding, and M9 and NLS are mainly responsible for hnRNP nuclear retention.

The structure of hnRNPs usually includes RNA-binding/RNA recognition motifs and other domains/motifs related to cytoplasmic redistribution or the binding of nucleotide sequences (22, 23). Although hnRNPs share some similar structural features, they can be very different from each other (see **Figure 1**). Many members of hnRNPs possess RNA recognition motifs/RNA binding domains (RRMs/RBDs), while hnRNP E and hnRNP K possess specific RNA binding domains called K-homology domains (KH domains) (16). These structures identify and bind to specific RNA sequences, so different hnRNPs have distinctive sequence affinities. For example, the RRM1 of hnRNP A2/B1 recognizes adenine-guanine–guanine (AGG) motifs, and its RRM2 recognizes uridine-adenine-guanine (UAG) motifs (24). Most hnRNPs are confined within the nucleus, while a few others can shuttle between the cytoplasm and nucleus (18, 25). Several structures are responsible for their localization rearrangement. Some hnRNPs contain a nuclear localization sequence (NLS), which is in charge of nuclear import (26). The other sequence that mediates the hnRNPs nuclear import/export is the M9 sequence (27). However, more information on the mechanisms by which hnRNPs are exported from the nucleus to the cytoplasm remains to be defined. It is also worth mentioning that the abundance of hnRNPs is distinctive in different organisms (for example, hnRNP C was identified to be highly expressed in the neurons and testicles of mice but not detectable in the lung or pancreas) (28). In addition to binding RNA, hnRNPs are also associated with DNA biogenesis as they are involved in DNA replication, damage repair and telomere functioning (29). For instance, it has been shown that hnRNP K can modulate neurotransmitter gene biosynthesis and participate in activation-induced cytidine deaminase-mediated antibody diversification (30, 31).

HnRNPs are involved in many steps of viral infection process, including replication, translation, the switch of translation to replication, as well as virion release (19, 32, 33). For example, the SARS-CoV-2 N protein can partition into liquid condensates with hnRNP A2 and hnRNP P to promote viral replication (19). The negative-stranded RNA of poliovirus (PV) could interact with hnRNP C to enable positive-stranded RNA synthesis (32). During enterovirus 71 (EV71) infection, hnRNP A1 can bind to viral internal ribosome entry site (IRES), which leads to enhanced IRES-mediated translation, and hnRNP K interacts with stem-loops I, II, and IV to participate in viral replication (34, 35). Some viruses could take advantage of hnRNPs by rearranging these proteins from the nucleus to the cytoplasm (36, 37). With positive-stranded RNA viruses replicate in the cytoplasm, distributed hnRNPs are able to interact with viral proteins or RNA to either assist or hinder virus multiplication (1, 36, 37). Therefore, discussing the interactions between viruses and hnRNPs improves our understanding of the molecular mechanisms of viral attacks on host cells and the strategies through which our bodies resist these invasions (38).

## Roles of heterogeneous nuclear ribonucleoproteins in the positive-strand virus life cycle

### HnRNP A/B

The four paralogues of hnRNP A/B proteins are hnRNP A1, A2/B1, A3 and A0, and all of them were reported to have several isoforms except for A0. The structures of hnRNP A/B proteins are highly conserved among each other and they normally locate at the nucleus (39). Another study suggested that although hnRNP A/B colocalized with spliceosomal complexes within the nucleus, hnRNP A1 was abundant at the membrane of the nucleus while A2/B1 and A3 accumulated in perinucleolar areas (40). HnRNP A/B are responsible for RNA splicing, trafficking and mRNA translation regulation (both Cap-dependent and IRES-dependent). Besides, hnRNP A1 and A2/B1 also possess DNA-binding ability (39).

Among these subgroup proteins, hnRNP A1 is one of the most abundant and ubiquitously expressed proteins (27). HnRNP A1 contains an unwinding protein 1 (UP1) domain comprising two RNA-recognition motifs (RRM1 and RRM2) in the N-terminus followed by specific motifs, an RGG box, a prion-like domain and a nuclear-shuttling sequence called the M9 sequence in the C-terminus (26, 41). UP1 and the RGG-box affect the ability of hnRNP A1 to unfold DNA G-quadruplexes, and the prion-like domain is closely related to stress granule assembly (41, 42). HnRNP A1 shuttles rapidly between the cytosol and the nucleus, and its M9 is vital for its import back into the nucleus. A study revealed that TMG-induced O-linked N-acetylglucosaminylation reinforces hnRNP A1 nuclear localization and that sorbitol-induced phosphorylation of hnRNP A1 results in its cytoplasmic accumulation (43). Notably, although hnRNP A1 is expressed in most tissues, it was identified to be most abundant in neurons of the central nervous system (28). HnRNP A2/B1 is crucial to oligodendrocyte and neural mRNA trafficking (44).

Some coronaviruses were reported to be associated with hnRNP A1 (45–51). An early study suggested that the nucleocapsid protein (N protein) of SARS coronavirus had a high affinity with hnRNP A1, and the protein-protein interaction requires 161-220 aa of SARS coronavirus N protein and 203-320 aa of hnRNP A1 (45). It was also suggested that hnRNP A1 might participate in the switch from viral translation to replication because N6-methyl adenosine (m6A) marked SARS-CoV-2 RNA recruit hnRNP A1 and enhance viral genome transcription while suppressing translation. And this interaction could be inhibited by 3-Deazaneplanocin A (DZNep) (46). And During SARS-CoV-2 infection, the cellular location of hnRNP A2/B1 is rearranged by NSP1, leading to restrained immune response and enhancing infection by SARS-CoV-2 and β-coronavirus, but the mechanism by which this occurs remains to be explained (36). And a recent study pointed out that hnRNP A2/B1 could associate with SARS-CoV-2 RNA to promote viral replication, which could be targetable for antiviral drugs (52).

**Figure 2:**
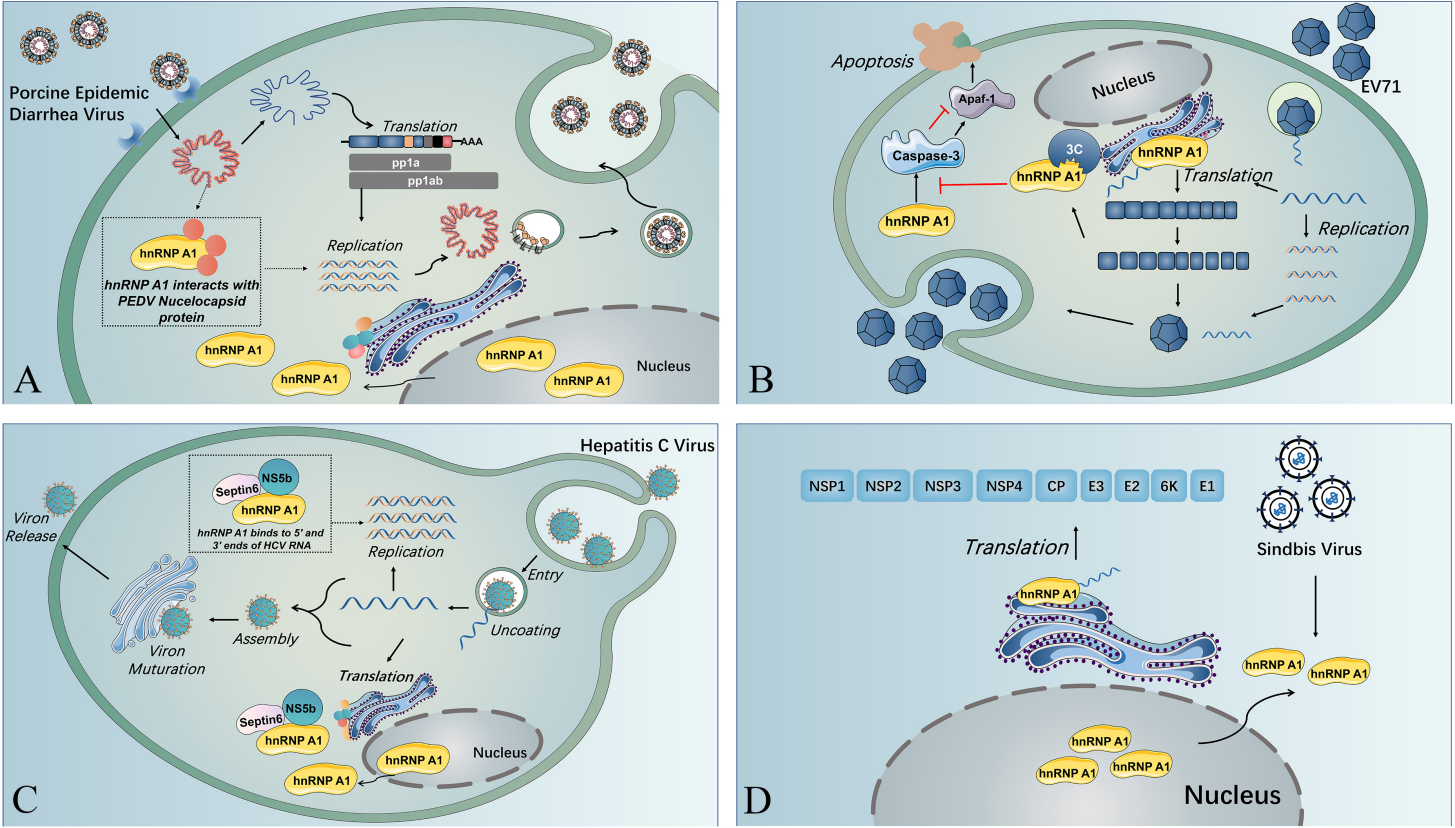
The multiple functions of hnRNP A1 in viral life cycles. **(A)** Nuclear translocation of SV induces cytoplasmic retention of hnRNP A1, and hnRNP A1 binds to the 5’ UTR of SV RNA, resulting in enhanced viral translation. **(B)** HnRNP A1 interacts with the nucleocapsid of PEDV and facilitates PEDV replication near the nucleus. **(C)** HnRNP A1 binding to the 5’ UTR and 3’ UTR of HCV RNA and forming a complex with septin 6 and NSb5 induces the cyclization of HCV RNA and reinforces HCV RNA replication. **(D)** HnRNP A1 could bind to Apaf-1 mRNA to promote Apaf-1 translation and then upregulate the expression of caspase-3, resulting in cell apoptosis and virion release. EV71 3C protease could splice hnRNP A1 and abolish its capacity to bind to Apaf-1 mRNA and downregulate caspase-3 expression, guaranteeing sufficient virus replication before virion release.

HnRNP A1 interacts with the porcine epidemic diarrhoea virus (PEDV) N protein to promote viral replication, and inhibition of hnRNP A1 could result in reduced virus copy numbers of different strains of PEDV in CCL81 cells (47) (see **Figure 2A**). Despite the lack of evidence, hnRNP A1 was hypothesized to facilitate PEDV replication through binding to the 5’ end sequence and intergenic IG sequence, which is required for coronavirus optical transcription of nested subgenomic mRNA (47). Interestingly, the hnRNP A1 level was downregulated during PEDV YN144 strain infectin, a finding different from that in cells infected with the YN13 strain, where the hnRNP A1 levels were not remarkably changed. This phenomenon was presumed to be related to the weaker virulence of YN144 (48). Mouse hepatitis virus (MHV) infection could result in cytoplasm retention of hnRNP A1 and binding of hnRNP A1 to transcription regulation areas of MHV negative-stranded RNA (49). Interestingly, C-terminal deletion of hnRNP A1 inhibited MHV replication, while full-length hnRNP A1 reinforced MHV replication (50). hnRNP A1 was also detected to interact with MHV N proteins in the cytoplasm, but the effect of this interaction during MHV infection remained unexplored (51). As mentioned above, the N protein of SARS-CoV-2, PEDV and MHV could interact with hnRNP A1, and the interaction favours the virus replication (47).

HnRNP A1 also affect the replication of viruses from other virus families (20, 53). HnRNP A1 usually affects the viral translation by associating with the IRES within viral RNA (20, 53, 54). The IRES trans-acting factor (ITAF) activity of hnRNP A1 could be regulated through posttranslational modifications (PTMs). HnRNP A1 PTMs are recognized by different viruses to modulate their IRES-dependent translation (55). HnRNP A1 acts as an ITAF with EV71 IRES to regulate IRES-dependent translation, and hnRNP A2 shows a similar function during EV71 infection. Furthermore, EV71 infection stimulates the phosphorylation of p38 mitogen-activated protein kinase (MARK), which induces the cytoplasmic relocalization of hnRNP A1 and induces IRES-mediated viral protein translation (56). A study indicated that EV71 translation could be restrained by inhibition of hnRNP A1 shuttling from the nucleus to the cytoplasm through the use of an inhibitor (SB203580) that can inhibit p38 MAPK (57). The association of hnRNP A1 and A2 on EV71 IRES was demonstrated to be inhibited by a dietary flavonoid called apigenin, and virus infection was downregulated when cells were given apigenin (53), which could be that apigenin target the glycine-rich domain of hnRNP A2, disrupting its multimerization and splicing activity (58). HnRNP A1 can trigger IRES-mediated translation of human rhinovirus (HRV) RNA and inhibit IRES activity of apoptotic peptidase activating factor 1 (apaf-1) mRNA. The binding of hnRNP A1 to the apaf-1 IRES hinders apaf-1 from hampering cell apoptosis and guaranteeing that the virus propagates sufficiently before releasing virions (59). The EV71 3C protease cleaves hnRNP A1, promoting apaf-1 translation and apoptosis and enables virus spreading (54) (see **Figure 2B**). Besides, hnRNP A1 could bind to the 5’-untranslated region (UTR) and 3’-UTR of the HCV genome, forming a complex with NS5b and septin 6 to promote viral replication (20) (see **Figure 2C**). In addition to IRES-mediated translation of RNA viruses, hnRNP A1 could also affect non-IRES-initiated translation, such as that of Sindbis virus (SINV). And during SINV infection, hnRNP A1 also undergoes retention in the cytoplasm, binding to the 5’ UTR of SINV RNA and promoting SINV translation, but the exact mechanism remains to be explored (60) (see **Figure 2D**).

HnRNP A2 has been confirmed to interact with Japanese encephalitis virus (JEV) NS5 by binding to the 5’ UTR of the negative-stranded RNA to enhance viral replication (61). Additionally, hnRNP A2 also binds to the 3’ UTR of DENV (62). And hnRNP A2 has been discovered to show RNA-binding activity similar to that of hnRNP A1 to MHV, modulating MHV RNA synthesis (63). During persistent Junín virus (JUNV) infection, not only was the location of hnRNP A/B rearranged to the cytoplasm, but the expression level of hnRNP A/B was also lowered than that under normal conditions (64). And when hnRNP A1 and hnRNP A2 were silenced, the replication of JUNV was significantly reduced, and JUNV infection caused the cytoplasmic distribution of hnRNP A1 but not hnRNP A2 (61, 65).

### HnRNP C

HnRNP C is a critical RNA-binding protein with functions in RNA expression, stability, mRNA splicing, nonspecific sequence exportation and 3’-end processing (66, 67). HnRNP C is predominantly located in the nucleus, and its expression level was upregulated in multiple tumours, including lung cancer, hepatocellular carcinoma, glioblastoma, melanoma and breast cancer (68–71). HnRNP C1/C2 consists of a RRM, a basic leucine zipper-like motif (bZLM), a NLS and an acid-rich C-terminal domain. There are 13 amino acid residues between RRM and bZLM in hnRNP C2, distinguishing it from hnRNP C1 (67). HnRNP C proteins can form C1_3_C2 tetramers in native hnRNP complexes (32, 66, 72).

Although HnRNP C1/C2 is normally located in the nucleus, its trafficking from the nucleus to the cytoplasm is observed during PV and RV infection (68). This relocalization may be attributed to either of the two mechanisms: the degradation of the nuclear pore complex (NPC) or the interaction with viral proteins and cellular proteins (66). The NPC forms a channel that allows macromolecules to shuttle between the cytoplasm and nucleus (69). Degradation of the NPC components Nup153 and p62 during RV or PV infection may be related to the inhibition of nuclear import pathways, resulting in cytoplasmic accumulation of hnRNP C1/C2 (70, 71).

HnRNP C can interact with PV RNA and proteins to stimulate viral RNA synthesis, as hnRNP C serves as an important component of RNP during PV infection-induced complex formation that promotes the initiation of positive-strand RNA synthesis (72). HnRNP C binds to both termini of virus negative-stranded RNA, forming a multimer that facilitates PV RNA synthesis. And C-terminal truncated hnRNP C1/C2 inhibits PV replication, suggesting that hnRNP C1/C2 associates with PV RNA through its C-terminus (72). During picornavirus infection, negative-stranded RNA is the template for viral replication, and the circulation of viral RNA is crucial for efficient replication, so the hnRNP C stabilizing interaction between the 5’-UTR and 3’-UTR of negative-stranded RNA contributes to viral replication (32).

Other members of the *Picornaviridae* family may show the same regulatory action due to the highly conserved sequence within the IRES (73). During Coxsackie B virus (CVB3) infection, hnRNP C1/C2 could bind to the 5’ UTR of virus RNA and replace polypyrimidine tract-binding protein (PTBP, or hnRNP I) and bind to stem-loop V in the CVB3 IRES, inhibiting the translation of CVB3. And it could mediate the translation-replication switch without the help of CVB3 3CD (73, 74). Interestingly, hnRNP C1/C2 exhibits a higher affinity for negative-stranded viral RNA than positive-strand viral RNA, although positive-strand viral RNA outnumbered negative-stranded viral RNA (74). During CVB3 infection, the positive-stranded/negative-stranded viral RNA ratio altered under the control of hnRNP C1/C2 (74).

In addition to interacting with picornavirus RNA, hnRNP C1/C2 has been discovered to bind to precursors of PV 3CD, P2 and P3 precursors, which likely recruit 3CD to the replication complex/replication organelle (RC/RO) (68). The RC/RO is a unique structure that forms in positive RNA virus-infected cells and contains several viral proteins and host factors required for efficient replication of viral RNA (75). And how the association of hnRNP C1/C2 and RC/RO contribute to the viral replication require further investigation.

Multiplication of a member of *Flaviviridae* family is also reported to be regulated by hnRNP C1/C2 (76). Knockdown of hnRNP C1/C2 using specific siRNA, and the hnRNP C1/C2 knockdown cells were less infected by DENV compared to normal cells. And the viral RNA level and relative expression level of viral proteins declined while hnRNP C1/C2 is knocked down (but not through directly resisting viral translation). Notably, the supernatant virus titers were also lowered in hnRNP C1/C2 knockdown cells (77). HnRNP C1/C2 can also interact with the DENV NS1 protein, but whether it affects DENV infection remains unknown, and further exploration is required (76).

### HnRNP D

Due to alternative exon splicing, four protein isoforms of hnRNP D (also known as AU-rich element RNA-binding protein 1, AUF1) have been identified and named based on their molecular weight: p37^AUF1^, p40^AUF1^, p42^AUF1^ and p45^AUF1^. All these isoforms contain two RRMs and a glutamine-rich (Q-rich) motif (16). Isoforms p37^AUF1^ and p40^AUF1^ have a nuclear import signal, while p42^AUF1^ and p45^AUF1^ have a nuclear export sequence within exon 7, while the two smaller isoforms lack the sequence (78). All four isoforms of hnRNP D were reported to be mainly located in the nucleus, but they could shuttle between the cytoplasm and nucleus in a transcription-dependent manner. It was also suggested that the interaction between the smaller two isoforms and two larger isoforms might contribute to the shuttle function of hnRNP D. HnRNP D is an extensively studied AU-rich-binding protein predominantly responsible for rapid mRNA degradation. In addition, hnRNP D regulates the stabilization of ARE-mRNAs and the transcription of certain genes (79).

Among the hnRNP D isoforms, p45^AUF1^ significantly promotes the replication of several members of the *Flaviviridae* family, including Zika virus (ZIKV), West Nile virus (WNV), DENV and HCV (80). It was reported that p45^AUF1^ could reinforce WNV RNA synthesis by inducing a structural shift of WNV RNA and enhancing the WNV RNA 5’- 3’ interaction by binding the AU-rich region of the WNV RNA 3’ UTR and destabilizing the 3’ stem structure of the 3’ CL of WNV RNA (81). The same research group reported that although hnRNP D is generally considered an AU-rich binding protein, the AU-rich sequence of WNV RNA was not required for p45^AUF1^-mediated WNV replication reinforcement *in vitro* but was necessary *in cellulo* (81). In addition to acting as an RNA chaperone for WNV RNA, p45^AUF1^ was also suggested to have an annealing function over WNV RNA, and the RNA chaperone activity is regulated by arginine methylation at the C-terminus of p45^AUF1^. The methylation of p45^AUF1^ mediated by methyltransferase PRMT1 remarkably increases p45^AUF1^ affinity to WNV RNA, thereby strengthening the binding ability of p45^AUF1^ to viral RNA and supporting efficient WNV RNA synthesis (82).

Similar to the function of hnRNP D during WNV infection, a later study demonstrated that p45^AUF1^ also destabilizes DENV and ZIKV RNA 3’ stem-loops as well as 5’ stem-loops to facilitate negative-stranded RNA synthesis by aiding in shifting viral translation to replication. As expected, depletion of p45^AUF1^ reduced DENV and ZIKV replication in human cells (80). The interaction of hnRNP D and HCV IRES facilitates viral translation (p45^AUF1^ had the strongest effect), and siRNA-mediated knockdown of hnRNP D remarkably downregulated viral replication (83). Encouragingly, HCV RNA can move from heavy polysomes to light polysomes when hnRNP D is reduced (83).

Unlike the roles of hnRNP D in the *Flavivirus* family, hnRNP D is predominantly a restriction factor of viral replication for enteroviruses (37, 84). All four isoforms were reported to bind to stem-loop IV of both PV and HRV, and the copy numbers of the viruses were increased in the absence of hnRNP D, suggesting that hnRNP D somehow limited the virus infection (85). HnRNP D could restrict PV and CVB3 replication by inhibiting viral RNA synthesis and IRES-driven translation, and the inhibition of hnRNP D on viral RNA synthesis is not due to interacting with the 3’ NCR of viral RNA or inducing viral RNA decay (86). Interestingly, EV71 translation is affected by hnRNP D but not EMCV RNA synthesis (86). Although cytoplasmic retention of hnRNP D was discovered in PV-, CVB3-, HRV-, EV71- and EMCV-infected cells, EMCV uses a different approach from other enteroviruses (37). And unlike other enterovirus 2A protein acting as a protease, the 2A protein of EMCV does not exhibit protease activity. However, it was indicated that Nup62 and Nup153 were targeted by both enterovirus 2A protein (through cleavage) and EMCV L protein (through phosphorylation), and thus, the nucleocytoplasmic transport feature of them was altered (70, 87). HnRNP D colocalizes with the 2A protein near the predicted replication complex in the cytoplasm in PV- and HRV16-infected cells (85).

**Figure 3:**
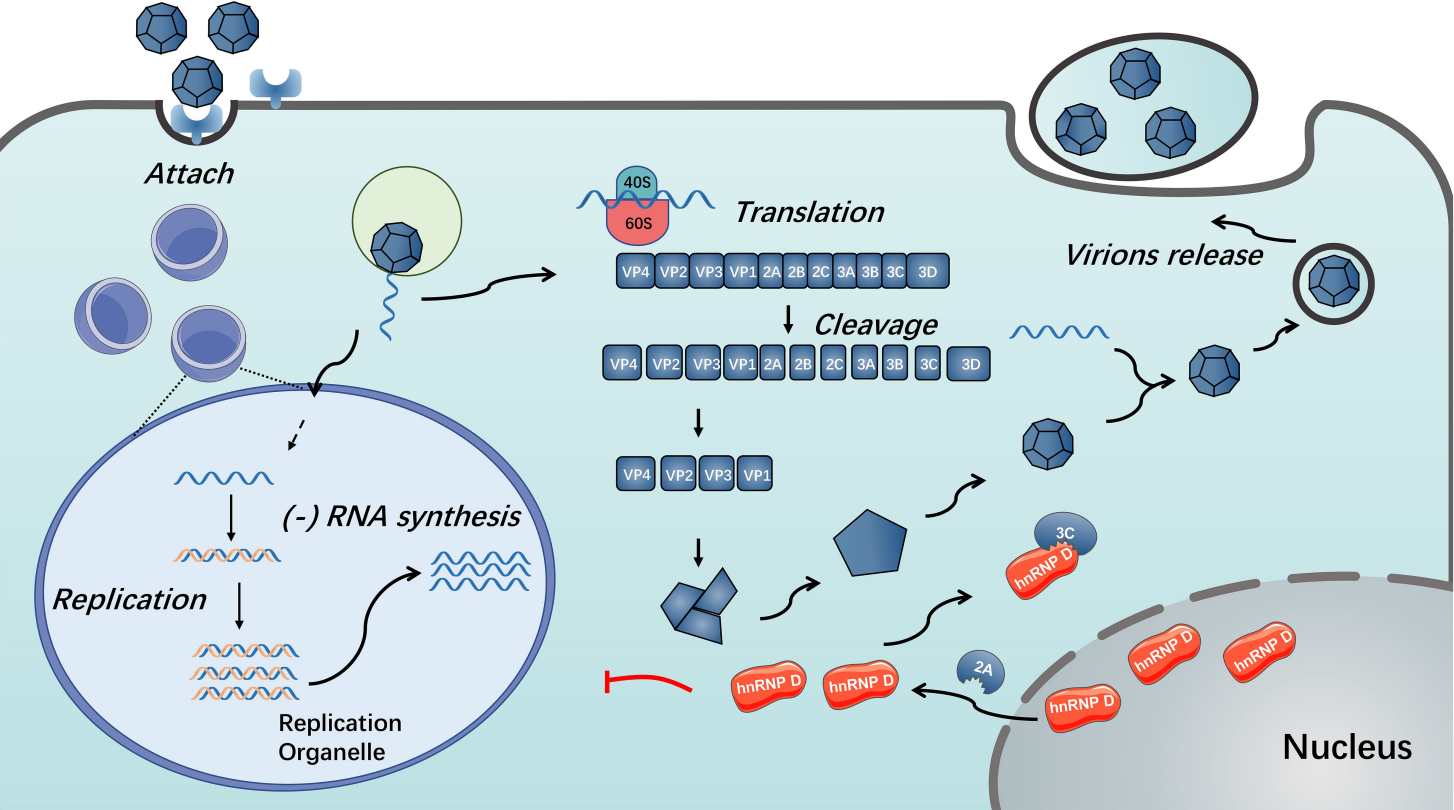
Functions of hnRNP D in enterovirus replication. During enterovirus infection, hnRNP D translocates from nucleus to cytoplasm in a 2A protein-dependent manner. The presence of hnRNP D could restrict enterovirus RNA replication. Enterovirus 3C/3CD could cleave hnRNP D and disable it from inhibiting virus RNA replication (85, 86).

In addition to changing the properties of the nuclear pore complex, the 3C/3CD of several enteroviruses blocks the restriction activity of hnRNP D by impairing their multiplication (86). HnRNP D was confirmed to be cleaved by 3C/3CD of PV, HRV and CVB3 (86). This cleavage results in disruption of hnRNP D binding to stem-loop IV of the viral RNA strand and thus resists restriction of hnRNP D on virus propagation, possibly caused by cleavage at the N-terminus to impair the dimerization of hnRNP D (85). Furthermore, because the CVB3 genome contains AU-rich sequences within the 3’ UTR, the cleavage of hnRNP D by CVB3 3CD can disrupt the binding of hnRNP D to the 3’ UTR of CVB3; thus, the stability of viral RNA is reinforced (84) (see **Figure 3**). Unlike the situation during enterovirus infection, there were not observable cleavage of hnRNP D was detected during EMCV infection (37). According to the studies mentioned above, the distinct behaviour of hnRNP D during EMCV infection might attribute to the alternative function of EMCV 2A protein (85–87).

Notably, hnRNP D was also reported to be recruited to stress granules (SGs) during high-dose infection with CVB3 and EV71 (88). Stress granules are complexes without membrane structures that form under stress pressure, such as viral infection, and can stall overall translation, including viral translation (89), so figuring out relationships of hnRNP D and viral RNA/proteins within SGs might reveal a mechanism that affect viral translation.

### HnRNP E

HnRNPs E1 and E2 are also known as poly(C)-binding protein-1 (PCBP-1) and PCBP-2. The remaining two members (hnRNP E3 and E4) are divergent from hnRNP E1 and E2 (16, 90). Unlike hnRNP E1 and E2, which are located in the nucleus, hnRNP E3 and E4 are identified to be predominantly in the cytoplasm, and are generally not considered as hnRNPs. And they all contain 3 KH domains (16). Each KH domain contains three α-helices and three β-folds and can target specific RNA and DNA. The sequences in KH domains are highly conserved, but sequences outside the KH domains vary. Both hnRNP E1 and E2 are highly expressed in the nucleus, exhibiting 89% similarity in sequence homology. However, the cytoplasmic roles of hnRNP E1 and E2 have attracted considerable attention because of their roles in alternative splicing, mRNA stability and translation. As an RNA chaperone, hnRNP E1 can unfold the secondary structure of the IRES, facilitating the binding of hnRNP I and recruitment of ribosomes to initiate translation (17, 90).

**Figure 4:**
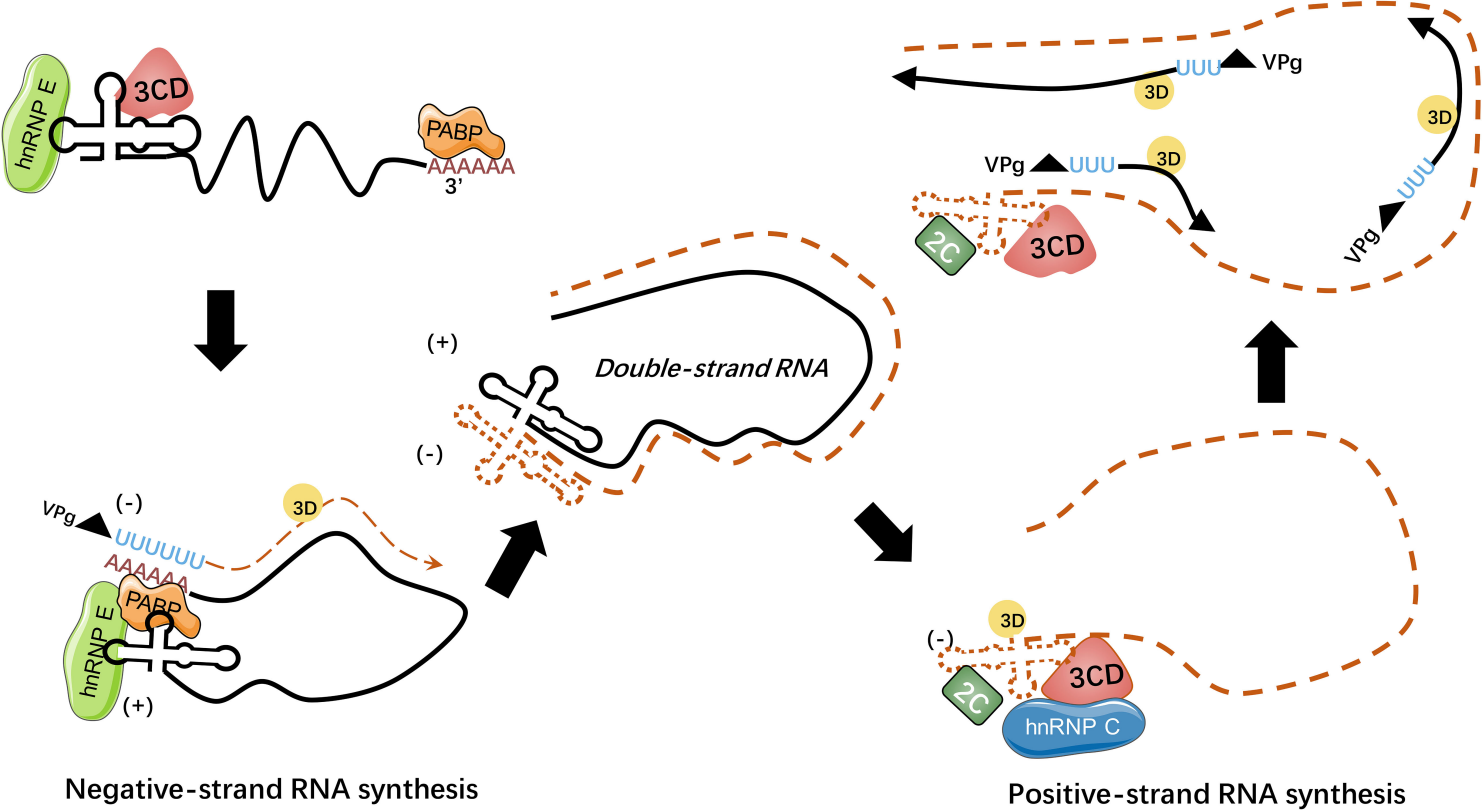
HnRNPs regulate picornaviral RNA synthesis. During picornavirus infection, viral genome circularizes through the interaction of hnRNP E-cloverleaf-3CD complex with PABP to initiate the synthesis of minus-strand RNA. The hnRNP E could bind to cloverleaf of positive-strand RNA with 3CD precursor while hnRNP C could bind to the minus-strand RNA with viral 2C ATPase to stabilize the cloverleaf structure. The processes of RNA replication rely on the interaction between hnRNPs and viral template RNA (98).

HnRNP E were predominantly reported to be associated with stem-loops of viral RNA, within cloverleaf or IRES (91–97). During PV infection, hnRNP E2 was confirmed to bind to stem-loop V of PV IRES and required for PV translation (91, 92). Both hnRNP E1 and hnRNP E2 bind to stem-loop IV and cloverleaf, and they form heterodimers to interact with PV RNA to reinforce viral translation (93). Another study pointed out that although hnRNP E1 and E2 both have the capacity to bind to PV cloverleaf and stem-loop IV, these two isoforms have different affinities: hnRNP E2 was identified to have a much stronger capacity to bind to PV stem-loop IV than hnRNP E1, while the binding ability of PV cloverleaf was similar (97). Furthermore, hnRNP E binds to stem-loop B of PV RNA cloverleaf to remarkably strengthen PV 3CD binding to stem-loop D of PV RNA cloverleaf, and they form a hnRNP E-RNA-3CD ternary complex (93) (see **Figure 4**). Together, hnRNP E and PV 3CD can modulate the switch from viral translation to replication (94). It was assumed that cleavage of hnRNP E by PV 3C/3CD mediates the viral translation-replication shift because the cleavage between the KH2 and KH3 domains results in the truncated hnRNP E that lacks the KH3 domain, with intact function in replication but impaired function in translation (95). Interestingly, a study indicated that PV 3CD binds to cloverleaf stem-loop D of PV RNA to rearrange hnRNP E from binding PV cloverleaf to stem-loop IV to some degree, while the importance of this activity remained unspecified (96).

During EV71 infection, hnRNP E1 binds to stem-loops I and IV within the 5’-UTR after being recruited to the viral membrane-associated complex to facilitate replication (99). APOBEC3G (A3G), a broad-spectrum antiviral factor, competitively binds to the EV71 5’-UTR to restrain the interaction between the 5’-UTR and hnRNP E1 and inhibiting viral replication and protein synthesis (100). Whether A3G could inhibit 5’ UTR-hnRNP E1 interaction of other viruses awaits future studying.

This KH domain-dependent hnRNP E-viral RNA interaction could protect PV RNA from 5’ exonuclease and maintain viral RNA stability of coxsackieviruses, echoviruses, and rhinoviruses (101). Stabilized viral RNA is essential not only for viral polysome formation but also for efficient viral translation and replication (101, 102). The interaction of the hnRNP E2 KH3 domain and PV cloverleaf could stimulate PV translation, along with the interaction between PV 2A and poly(A) tail (103). CVB3, another enterovirus member, was also reported to interact with hnRNP E (104). HnRNP E2 was identified to interact with cloverleaf and IRES IV of CVB3 RNA, with its KH1 domain binding to subdomain C of IRES IV and the KH3 domain binding to subdomain B of IRES IV (105). Similar to the findings in PV-infected cells, hnRNP E binding to the cloverleaf of CVB3 RNA to facilitate the synthesis of viral subgenomic negative-stranded RNA (106).

Calicivirus IRES was slightly distinct from type I IRES of PV, it constituted an important GNRA tetraloop in subdomain d10c. This tetraloop was identified to bind to the KH1 and KH2 domain of hnRNP E2, along with another subdomain d10b within IRES binding to the KH3 domain of hnRNP E2. And the KH2 domain of hnRNP E2 is required for efficient calicivirus replication initiation (107). HnRNP E1 and E2 also interact with HCV 5’ UTR RNA, but their roles during HCV infection require more research to specify (108). A later study demonstrated that hnRNP E2 could bind to HCV IRES to promote viral translation. hnRNP E2 partially localize to the detergent-resistant membrane fraction and associate with HCV nonstructural protein NS5 to facilitate viral replication by circularizing the HCV genome within the HCV replication complex (109). Porcine reproductive and respiratory syndrome virus (PRRSV) was also associated with hnRNP E1 and E2 (110). HnRNPs E1 and E2 were discovered to interact with the PRRSV RNA 5’ UTR and nonstructural protein 1β (nsp1β) to be involved in PRRSV genome replication and transcription and colocalize with the viral replication and transcription complex in the cytoplasm (110). Another study confirmed that the nsp1β-hnRNP E2 interaction requires the C-terminal extension (CTE) domain and C-terminal papain-like cysteine protease domain (PCPβ) domain of PRRSV nsp1β and the KH2 domain of hnRNP E2, and its putative mechanism for modulating viral translation and replication is through controlling the translation-replication switch (111). Both hnRNP E1 and hnRNP E2 can interact with the P protein of vesicular stomatitis virus (VSV) and antagonize viral infection by reducing viral gene expression (112).

### HnRNP I

HnRNP I, also known as PTBP, regulates splicing by binding to polypyrimidine stretches at a branch point upstream of exons. Similar to the structure of hnRNP L, hnRNP I composed of four RRMs. Both hnRNP L and hnRNP I participate in RNA-related biological processes, including mRNA stabilization, pre-mRNA and translation. Both PTBP1 and PTBP2 can interact with CU repeats to repress nonconserved cryptic exons (113).

During positive single-stranded RNA virus infection, hnRNP I can regulate viral IRES-dependent translation and viral replication (114–117). As for picornaviruses, the IRES-mediated translation of EMCV and FMDV can be enhanced by hnRNP I (114, 115). More specifically, it has been shown that hnRNP I can bind to the FMDV IRES, forming an initiation complex with 48S and 80S (115). HnRNP I has also been shown to bind to the 3’ terminus of HCV RNA and support viral replication (116, 117). However, the role of hnRNP I during HCV infection is controversial; some articles suggest that hnRNP I promotes HCV translation and replication, while several articles have opposite opinions (118–122). A small-molecule compound, 6-methoxyethylamino-numonafide (MEAN), can inhibit HCV replication by hampering the expression level and redistribution of hnRNP I (123). Interestingly, during PV (sabin strain)-infection, an isoform of hnRNP I that is specifically expressed in neurons was identified to have a different function. Although neuron-specific hnRNP I shares 70% of the amino acids, it failed to rescue viral translation in hnRNP I-knockdown cells (124).

### HnRNP K

HnRNP K is a versatile RNA-/DNA-binding protein that is involved in multiple fundamental processes of gene expression and signalling, including chromatin remodelling, RNA splicing, mRNA stability, transcription and translation (125). Furthermore, hnRNP K is critical for cellular DNA damage repair and tumorigenesis (126). Similar to hnRNP E, hnRNP K contains three KH domains (KH1, KH2 and KH3), a K-interaction (KI) domain, a NLS, a nuclear shuttling domain (KNS), a proline-rich domain and an interactive region with a C-terminal kinase (cKBR) (125).

During HCV infection, hnRNP K was identified to interact with the HCV core protein and 5’ UTR of HCV RNA (127, 128). It was confirmed that amino acids 1-155 of the HCV core protein and amino acids 250-392 of hnRNP K (consist of 3 proline-rich domains) were responsible for the binding (127). Although how the modulation occurs remained unclarified, binding of hnRNP K to stem-loop I within the 5’ UTR of HCV RNA could aid HCV replication. Notably, hnRNP K was partially rearranged from the nucleus to the cytoplasm to colocalize with NS5A, a viral protein that is related to HCV replication complex formation (128). Interestingly, miR-122, as a highly expressed microRNA in livers, was also confirmed to bind to hnRNP K. And the interaction between miR-122 and hnRNP K would increase the stability of miR-122 and may possess the capacity to modulate HCV replication (129). Another study pointed out that hnRNP K binds to positive-stranded RNA of HCV and is recruited to lipid droplets to suppress HCV particle production, possibly by restraining the genome from packaging into virions without impairing viral replication, but viral RNA-hnRNP K interaction or downregulation of viral particles producing was not found during DENV infection (130). However, a study indicated that hnRNP K could affect viral multiplication and release by associating with vimentin and DENV NS1 because disruption of vimentin reduced nuclear expression of hnRNP K and downregulated virus titers of cell-associated DENV and culture supernatant (33). A similar phenomenon was observed during JEV infection, and JEV NS1 also interacted with vimentin and hnRNP K to support viral propagation (131).

HnRNP K was suggested to bind to the IRES of both EV71 and FMDV RNA, although the binding sites of hnRNP K on the IRES were slightly different (34, 132). Stem-loop I, II and IV of FMDV RNA were determined to interact with hnRNP K, and this interaction may result in enhanced viral RNA synthesis (34). During FMDV infection, hnRNP K binds to domains II, III and IV of FMDV IRES and thus inhibits FMDV translation by replacing PTB, which functions as an ITAF to promote FMDV translation (132). Notably, FMDV 3CD protease could cleave hnRNP K at Glu-364, producing two cleavage products, hnRNP K (aa 1-364), remining a minor restriction on FMDV IRES-mediated translation, and hnRNP K (aa 364-465), which was confirmed to promote FMDV propagation (132). HnRNP K could also be cleaved by PV and CVB3 3C protease, although hnRNP K could benefit the virus infection (133).

For JUNV, hnRNP K was confirmed to be recruited from the nucleus to the cytoplasm during infection to favour virus propagation (65). SINV nsP1, nsP2 and nsP3 were found to be coimmunoprecipitated with hnRNP K, and nsP2 could colocalize with hnRNP K in infected cells (134). HnRNP K could also interact with Chikungunya virus (CHIKV) nsP2 and capsid, and knockdown of hnRNP K induces lower virus copies, suggesting that it may play an essential role in CHIKV multiplication (135). HnRNP K is also required for VSV infection by several mechanisms (136). First, hnRNP K suppresses the apoptosis of VSV-infected cells, promoting cell survival for efficient viral propagation (136). Notably, hnRNP K restricts the expression of T-cell-restricted intracellular antigen isoforms 1a and 1b (TIA1a and TIA1a), both of which can suppress VSV replication. Additionally, hnRNP K maintains the level of cellular proteins that are required for VSV infection, such as the alanine deaminase-like (ADAL) proteins GBF1 and ARF1 (136).

### HnRNP L

Similar to other members of hnRNPs, hnRNP L is involved in mRNA stabilization, mRNA transportation, pre-mRNA splicing and IRES-mediated translation. HnRNP L was first identified as a member of the hnRNP family. In particular, it has been reported that hnRNP L-directed RNA switches regulate the stress-dependent translation of vascular endothelial growth factor A (VEGFA) and promote cell apoptosis (137). Furthermore, hnRNP L mediates cryptic exon repression by acting as a splicing factor and utilizing CA-rich elements (138). HnRNP L can also interact with other hnRNP family members, including hnRNP K, hnRNP I and hnRNP E2 (139, 140). HnRNP L has four consensus RRM domains, and although it is primarily distributed in the nucleus, it can be localized to the cytoplasm under hypoxic conditions (137).

HCV could recruit cellular eukaryotic initiation factors (eIFs) and ITAFs to the IRES elements, initiating and modulating translation through a complicated network of RNA–protein and protein–protein interactions and the contact between the 5’- and 3’-ends of the viral genome (21). HnRNP L specifically binds to the HCV IRES, promoting viral translation. And HCV IRES-mediated translation enhanced by hnRNP L could be blocked by an RNA aptamer specific for hnRNP L (21). In addition, HCV infection mediates the coprecipitation of hnRNP L with NS5A and increases the amount of hnRNP L associated with viral replication complexes (141). Depleting hnRNP L impairs viral replication and attenuates viral yield but does not affect HCV IRES-driven translation (141). FMDV IRES can specifically bind hnRNP L, negatively regulating viral replication in a manner that differs from IRES-dependent translation. Because hnRNP L could interact with FMDV 3D^pol^ in the presence of FMDV RNA, it was speculated that hnRNP L inhibits viral RNA synthesis in the replication complex (142). And for the limited amounts of studies on how hnRNP L affect positive singe-stranded RNA viruses life cycle (21, 141, 142), more investigations are needed to explain its function during virus infection.

### HnRNP M

As a component of the spliceosome complex, hnRNP M (or CEAR) is abundant in the nucleus and comprises four different isoforms generated by alternative splicing. The four isoforms constitute three RRMs with shifting locations (29, 143). HnRNP M is a critical splicing regulatory protein for some receptors with divergent physiological functions, such as fibroblast growth factor receptor 2 (FGFR2) and dopamine D2 receptor (D2R) (144, 145). Recent studies have revealed well-documented roles for hnRNP M in cancer metastasis, muscle differentiation and innate immune gene expression (146–148).

HnRNP M silencing can increase the replication of SINV, CHIKV and Semliki Forest virus (SFV), indicating hnRNP M could impede virus infection. And it is worth mentioning that hnRNP M, hnRNP C and hnRNP E1 colocalize with viral replicases in the cytoplasm (149). In contrast, knockdown of hnRNP M and hnRNP F significantly decreased DENV production, indicating the proteins are required for efficient viral production (150).

A subsequent study reported that loss of hnRNP M results in hyperinduction of a cohort of inflammatory and antimicrobial genes in VSV-infected macrophages, enhancing macrophage antiviral defences and controlling virus infection. This finding reveals that hnRNP M could restrain macrophage antiviral functions and positively regulate virus replication (151). During picornavirus infection, hnRNP M is cleaved by 3C/3CD of CVB3 and PV at position Q389/G390 between RRM2 and RRM3. Although the four isoforms of hnRNP M differ in length, all isoforms have this cleavage site (152). In addition, hnRNP M and/or its cleavage products were identified to facilitate protein synthesis and replication of PV, but they were not required for PV IRES-mediated translation or viral RNA stability maintenance (152).

HnRNP M also associate with innate immune pathways to regulate virus infection (148, 153). When retinoic acid-inducible gene-I (RIG-I)-like receptors (RLRs) recognize the viral genome, and the innate immune response is triggered against invading pathogens (153). During virus infection, hnRNP M can interact with RIG-I and MDA5 in a viral infection-dependent manner and negatively regulate the induction of antiviral genes triggered by Sendai virus (SeV) or EMCV (153). Moreover, hnRNP M could bind to viral RNA and weaken its binding affinity to RIG-I and MDA5, suggesting that hnRNP M could inhibit the innate antiviral response by antagonizing the sensing of viral RNA by RLRs (148).

### Other hnRNPs

Unlike other hnRNP proteins, hnRNP F and H appear to bind poly(G)-rich tracts, whose RRMs are not conserved and described as quasi-RRMs (qRRMs). In addition to regulating the maturation and posttranscriptional processing of pre-mRNA, hnRNP H/F protein was recently found to localize to stress granules in response to cellular stress. Although recognizing similar sequences, hnRNP F was upregulated, while hnRNP H and H2 were significantly down-regulated during Nipah virus infection (154). With 2D-gel electrophoresis and MALDI-TOF analysis, hnRNP H was identified to regulate DENV multiplication by affecting TNF-α production (155). HnRNP H was also reported to interact with the NS1 protein of DENV, aiding viral propagation, although the exact mechanism still remained unclear (156). The hnRNP U (also known as scaffold attachment factor-A, SAF-A), a key RNA-binding protein in processing newly transcribed RNA and chromatin organisation in interphase, was identified to interfere induction of some antiviral immune genes during VSV infection, and it may result from VSV-induced cleavage of hnRNP U which depleted C-terminal RGG domain, the RNA binding domain (157).

Other than interacting with positive single-stranded RNA viruses, hnRNPs could also affect life cycle of retrovirus (158). For example, hnRNP A1, AB, H and F were identified as HIV splicing factors that regulate HIV-1 splicing (158, 159). The splicing of HIV RNA increases the coding potential of the viral genome and controls viral gene expression. HnRNP A1, Q, K, R and U can bind to Rev protein specifically, which is a significant regulator in the HIV replication cycle. The knockdown of hnRNP A1, Q, K and R has a negative impact on HIV replication, while knockdown of hnRNP U can increase viral production (160). It is worth noting that the N-terminal 86 amino acids of hnRNP U could downregulate HIV mRNA transcription in the cytoplasm (139). HnRNP associated with lethal yellow (RALY), which shares a high sequence similarity with hnRNP C, regulates the expression of the HIV coreceptor CCR5 by binding to its 3’UTR (161). Thus, hnRNP proteins modulate HIV-1 gene expression by a series of multiple mechanisms. Using a proteomic strategy to define polysome specialization during RNA virus infection, hnRNP R has been identified as a novel ITAF recruited by PV for translation (162). HnRNP P (also known as FUS/TLS) directly inhibits the transcription and translation of CVB3 by inducing the formation of SGs, the production of IFN-I and inflammatory cytokines (163). Because of their key roles in the regulation of gene expression, it is not surprising that hnRNP proteins are involved in viral infection. These hnRNP proteins directly or indirectly influence viral translation and replication.

## Conclusion

Despite their diversity in structure (from hnRNP A1 to U), hnRNPs are involved in multiple cellular processes, including pre-mRNA processing, mRNA transport, regulation of translation, and controlling miRNAs (164, 165). To date, it has become clear that hnRNPs are crucial players in the cancer and neurodegenerative disease, and they are also established as playing either antiviral or pro-viral roles (44, 166).

**Figure 5:**
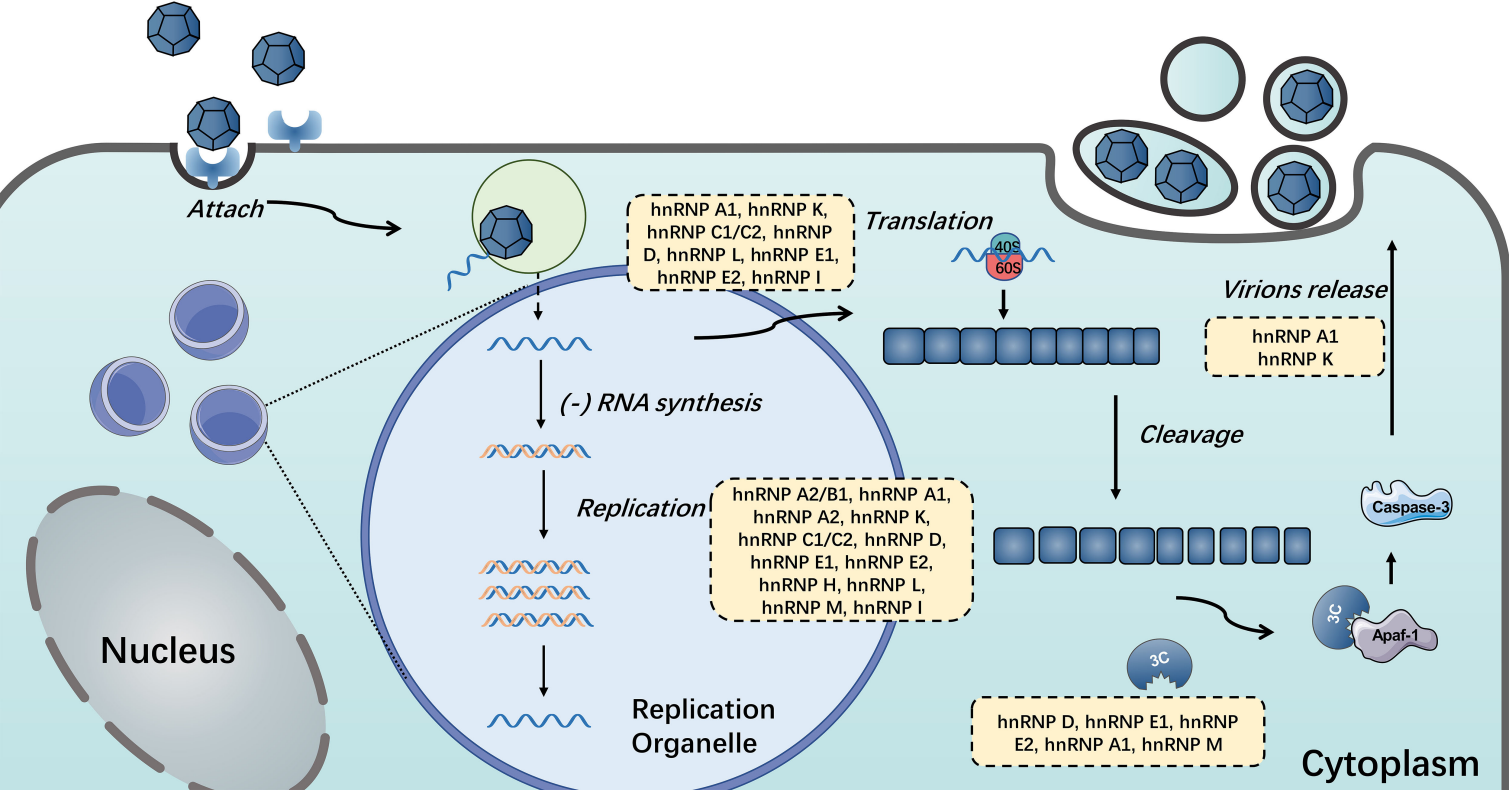
HnRNPs in the positive single-stranded RNA virus life cycle. HnRNPs play important roles in the life cycle of single-stranded RNA viruses, including viral translation, replication, the switch of translation to replication and the release of mature virions. The hnRNPs in yellow boxes were discovered to participate in these processes.

Given their diverse and important functionalities, it is not at all surprising that many hnRNPs have been linked either directly or indirectly to viral replication and pathogenesis. Normally, hnRNP I involves in pre-mRNA splicing, IRES-dependent translation initiation, RNA polyadenylation, transportation and stability, and cell differentiation (164). During infection, hnRNP I could act as an ITAF for HRV and FMDV IRES, which stimulates and controls viral translation (114, 115). Similar to hnRNP I, many hnRNPs could be manipulated by single-stranded RNA viruses *via* interacting with viral RNA or proteins to aid their life cycle proceeding (46, 74, 81, 91, 114). And a few hnRNPs display distinctive effects when host cells are infected with different positive-stranded RNA viruses. Here, we summarize the functions of hnRNPs that participate in different stages of positive single-stranded RNA viruses (see **Figure 5**). Notably, most hnRNPs shuttle continuously between the nucleus and the cytoplasm, which means the localization of hnRNPs is also vital for virus infection (65, 85, 110, 111). Positive-stranded RNA viruses replicate in the cytoplasm, which is associated with virus-induced membrane structures (99). Many nuclear resident hnRNPs underwent cytoplasmic relocalization during viral infection, including hnRNP A1, hnRNP C, hnRNP D, hnRNP E, hnRNP K and hnRNP M (49, 70, 85, 110, 128, 149). In term of hnRNP A1, the best-known member of hnRNP family, can interact with multiple viral proteins or RNA and regulate their life cycles, including SARS, HCV, DENV, MHV, PEDV, EV71, JUNV and SINV (35, 46, 47, 50, 54, 64). Like hnRNP A1, the common effects of hnRNPs on positive single-stranded RNA viruses reported were promotion of viral replication or translation (55, 81, 98).

The positive single-stranded RNA viruses take advantage of hnRNP family for their sufficient proliferation. In the case of *Picornaviridae*, PV infection requires hnRNP C, hnRNP D, hnRNP E, hnRNP R, hnRNP K, hnRNP M and hnRNP I (72, 85, 95, 103, 124, 152, 162). Meanwhile, PV applies strategy to cleave hnRNP E, hnRNP K, hnRNP M and hnRNP I by viral 3C protease and abolish their binding capacity (96, 133, 152). And many other positive single-stranded RNA viruses and hnRNPs share this similar pattern (54, 133). Furthermore, some hnRNPs have been reported to interact with other host proteins to modulate viral propagation (20). HnRNP A1 could interact with HCV NSb5 and septin 6 to enhance viral RNA circulation and eventually reinforce viral replication (20).

This review focuses on the interactions between hnRNPs and positive single-stranded RNA viruses. Here, we compared and discussed the function of hnRNPs in regulating the activity of viral translation (see **Table 1**) *via* protein-RNA interaction during different viral infection. And it seems that hnRNPs particularly bind to IRES of virus RNA to achieve this (21, 53, 107). Although the importance of hnRNPS during single-stranded RNA virus infection are explored in some extent (45, 74, 83), the exact mechanisms by which these interactions affect viral life cycle are not fully understood. Investigations into the precise function of these hnRNPs in viral infection are likely to provide great mechanistic insights and potential therapeutic targets for these infectious diseases.

**Table 1:** A brief summary of the functions of hnRNPs in the positive single-stranded RNA virus life cycle.

HnRNP	Virus		Functions during positive single-stranded virus life cycle	Viral RNA/protein that harm interplay with	References
A/B	*Flaviviridae* *Picornaviridae* *Coronaviridae* *Arenaviridae* *Togaviridae*	HCV, DENV EV71, HRV SARS-CoV-2, PEDV, MHV JUNV SINV	Enhance virus RNA replication, enhance viral translation, modulate virions release	SARS-CoV-2 N protein, PEDV N protein, EV71 3C protein, IRES of EV71 RNA, 5’ UTR and 3’ UTR of HCV RNA, SARS-CoV-2 RNA	(45–52, 55–60)
C1/C2	*Flaviviridae* *Picornaviridae*	HCV, DENV PV, CVB3	Enhance virus RNA replication, enhance/inhibit viral translation, mediate switch of viral translation to replication	DENV NS1 protein, negative-strand RNA of PV, IRES of CVB3 RNA	(32, 72–74, 76, 77)
D	*Flaviviridae* *Picornaviridae*	HCV, WNV, ZIKV, DENV PV, CVB3. HRV, EMCV	Enhance viral translation, enhance virus RNA replication Inhibit virus RNA replication	IRES of HCV RNA, 3’ end and 5’ end of DENV RNA, WNV and ZIKV RNA, PV and HRV 3C protein, PV and CVB3 2A protein	(80–86)
E	*Flaviviridae* *Picornaviridae* *Rhabdoviridae* *Togaviridae*	DENV EV71, PV VSV SFV	Enhance viral translation Inhibit viral gene expressing	5’ CL of PV RNA, 5’ UTR of EV71 RNA, PV 3C/3CD protein, DENV core protein, VSV P protein	(91–97, 99–106)
I	*Flaviviridae* *Picornaviridae* *Hepeviridae*	HCV ECMV, FMDV, PV HEV	Enhance viral translation, enhance virus replication	IRES of EMCV RNA, 5’ UTR (IRES) of PV RNA, 3’ terminal of HCV RNA, PV 3C protein	(114–124)
K	*Flaviviridae* *Picornaviridae* *Arenaviridae* *Rhabdoviridae* *Togaviridae*	DENV, HCV EV71, FMDV JUNV VSV CHIKV, SINV	Enhance viral translation, enhance virus replication, virion assembly and release Inhibit viral protein synthesis	IRES of HCV and EV71 RNA, HCV core protein, DENV core protein, HCV NS3 and core protein	(33, 34, 127–134)
L	*Flaviviridae* *Picornaviridae*	HCV FMDV, EMCV, CVB3	Enhance viral translation, inhibit viral RNA replication Inhibit viral translation	RCs of FMDV and HCV, FMDV 3CD protein, CVB3 2A protein and 3C protein	(21, 137–142)
M	*Flaviviridae Picornaviridae* *Togaviridae* *Rhabdoviridae*	DENV PV, CVB3, EMCV SINV, SFV, CHIKV VSV	Enhance viral translation, enhance viral RNA replication, evade immune response inhibit virus replication	PV and CVB3 3C/3CD protein	(148–153)

## Author contributions

JW and DS conceived, designed and wrote this manuscript. MW and AC revised the manuscript. YZ, SM, XO, XZ, JH, QG, SZ, QY, YW, DZ, RJ, SC and ML provided ideas contributing to the structure of this manuscript. All authors listed contributed to the completion of the manuscript and reviewed and approved the final manuscript.

## Funding

This work was supported by the Applied Basic Research Programs of Science and Technology Department of Sichuan Province (2022NSFSC0080), the China Postdoctoral Science Foundation (2020M683651XB), the earmarked fund for China Agriculture Research System (CARS-42-17), China Agriculture Research System of MOF and MARA and the Sichuan Veterinary Medicine and Drug Innovation Group of China Agricultural Research System (SCCXTD-2020-18).

## Conflict of interest

The authors declare that the research was conducted in the absence of any commercial or financial relationships that could be construed as a potential conflict of interest.

## Publisher’s note

All claims expressed in this article are solely those of the authors and do not necessarily represent those of their affiliated organizations, or those of the publisher, the editors and the reviewers. Any product that may be evaluated in this article, or claim that may be made by its manufacturer, is not guaranteed or endorsed by the publisher.
